# Rhinovirus Suppresses TGF-β-GARP Presentation by Peripheral NK Cells

**DOI:** 10.3390/cells12010129

**Published:** 2022-12-28

**Authors:** Susanne Krammer, Zuqin Yang, Hannah Mitländer, Janina C. Grund, Sonja Trump, Susanne Mittler, Sabine Zirlik, Susetta Finotto

**Affiliations:** 1 Department of Molecular Pneumology, Friedrich-Alexander-Universität (FAU) Erlangen-Nürnberg, Universitätsklinikum Erlangen, 91054 Erlangen, Germany; 2 Department of Medicine 1, Friedrich-Alexander-Universität (FAU) Erlangen-Nürnberg, Universitätsklinikum Erlangen, 91054 Erlangen, Germany

**Keywords:** TGF-β, TGF-β RII, TGF-β RI, asthma, rhinovirus, NK cells

## Abstract

Asthma is a chronic airway disease whose exacerbations are often triggered by rhinovirus infection. TGF-β1 induces rhinovirus replication in infected cells. Moreover, TGF-β1 is a pleiotropic mediator that is produced by many immune cells in the latent, inactive form bound to the latency-associated peptide (LAP) and to the transmembrane protein glycoprotein A repetitions predominant (GARP). In this study we wanted to investigate the effect of rhinovirus infection on the TGF-β secretion and the downstream signaling via TGF-βRI/RII in peripheral blood mononuclear cells from control and asthmatic patients after rhinovirus infection ex vivo. Here, we found a significant upregulation of TGF-βRII in untouched PBMCs of asthmatics as well as a suppression of TGF-β release in the rhinovirus-infected PBMC condition. Moreover, consistent with an effect of TGF-β on Tregs, PBMCs infected with RV induced Tregs, and TGF-βRII directly correlated with RV1b mRNA. Finally, we found via flow cytometry that NK cells expressed less GARP surface-bound TGF-β, while cytokine-producing NK^bright^ cells were induced. In summary, we show that rhinovirus infection inhibits TGF-β release in PBMCs, which results in the activation of both Treg and NK cells.

## 1. Introduction

The chronic airway disease asthma is the 14th most important disease in the world in terms of the extent and duration of the disorder [1]. Especially during symptomatic episodes, asthmatic patients suffer from shortness of breath, wheezing and coughing that critically affect their quality of life. These symptomatic episodes, so called exacerbations, are often caused by airway infections. In over 80% of exacerbations, infections with rhinovirus are the main trigger [2,3]. In fact, a correlation between the seasonal patterns of upper respiratory infections and hospital admissions for asthma was demonstrated in the past [4]. In mild or moderate asthma, exacerbations can be controlled by combinational therapy of inhaled corticosteroids and long-acting β2-agonists [5,6], while severe asthmatics still suffer from exacerbations despite therapy [7].

Rhinoviruses (RV) are positive sense, single-stranded RNA viruses that are non-enveloped and belong to the picornavirus family [8]. There are three main serotypes for human rhinoviruses, which are HRV-A, -B and -C. Depending on the serotype, the virus enters the cell via entry receptors. For RV-A (e.g., RV1b) and RV-B, intracellular adhesion molecule-1 (ICAM-1) and the low-density lipoprotein-receptor (LDLR) are the main target. RV-C enters via the cadherin-related family member 3 (CDHR3) [9,10]. At the site of entry, the rhinovirus first infects airway epithelial cells causing the production of different antiviral interferons, as well as pro-inflammatory cytokines like IL-1, IL-6, IL-8, RANTES and others [11,12,13].

The transforming growth factor beta (TGF-β) is a cytokine that is known for its immunoregulatory and also pro-fibrotic properties that are critically involved in airway remodeling during asthma [14]. This dual property has been associated to the fact that TGF-β1 induces immunosuppressive T regulatory cells and together with IL-6 induces TH17-dominated immune response [15]. TGF-β is secreted in an inactive, latent form bound to the latency-associated protein (LAP). The LAP-TGF-β complex can be linked to glycoprotein A repetitions predominant (GARP), which is a transmembrane protein known to bind LAP-TGF-β on different cell types [16,17]. GARP expression on immune cells promotes tolerance, preventing inflammation in diseases like allergies. The mature TGF-β1 homodimer is released upon degradation of LAP. Activators of this process can be integrins like αvβ6 or αvβ8 integrin [18].

We previously reported that TGF-β1 might be retained intracellularly in rhinovirus-infected PBMCs from preschool children [19]. As rhinovirus is an important trigger for airway remodeling, we wanted to further investigate the immune response to rhinovirus infection in adult healthy controls and asthmatic individuals with a special focus on TGF-β.

## 2. Materials and Methods

### 2.1. Human Study AZCRA

**Figure 1:**
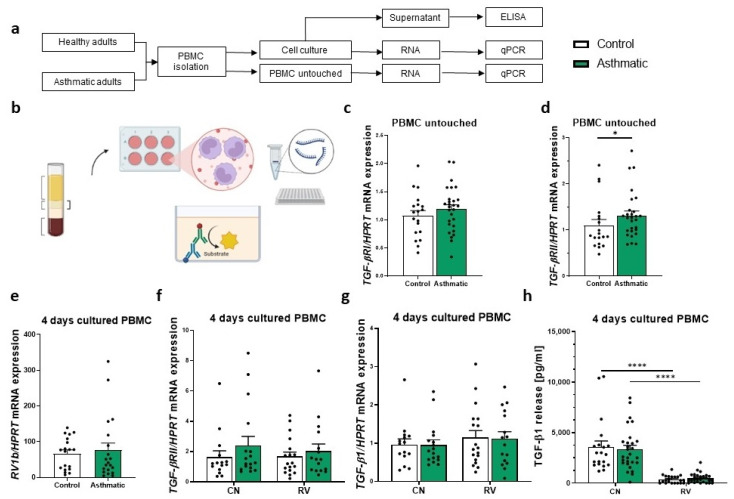
TGF-β response following rhinovirus infection in PBMCs. (**a**) Experimental design of the sample collection from healthy and asthmatic individuals. (**b**) Graphical abstract of the data collection. (**c**) Gene expression analysis of *TGF-βRI* expression on untouched PBMCs, normalized on the control group and the housekeeping gene *HPRT* (*n* = 19, 28). (**d**) Gene expression analysis of *TGF-βRII* expression on untouched PBMCs, normalized on the control group and the housekeeping gene *HPRT* (*n* = 19, 28). (**e**) Detection of *RV1b* load of RV-infected PBMCs via qPCR, normalized on *HPRT* mRNA expression (*n* = 19, 20). (**f**) Gene expression analysis of *TGF-βRII* expression in PBMCs, with control medium or infected with rhinovirus, and normalized on the control group and the house-keeping gene *HPRT* (*n* = 15, 18, 17, 16). (**g**) Gene expression analysis of *TGF-β1* expression on PBMCs with control medium or infected with rhinovirus, normalized on the control group and the housekeeping gene *HPRT* (*n* = 15, 18, 17, 16). (**h**) ELISA assay of TGF-β1 production in the supernatant of control medium and rhinovirus-infected PBMCs, activated in acidic condition before measurement, according to the supplier’s instructions (*n* = 19, 28). Data are shown as Mean+SEM. * *p* < 0.05, **** *p* < 0.0001.

We previously described the effect of in vitro rhinovirus infection on PBMCs in pre-school children with and without asthma recruited in the European PreDicta cohort in Erlangen [19]. To investigate rhinovirus infection in adult patients, we recruited healthy controls and asthmatics between 18 and 65 years for the new AZCRA study (Table 1, Figure 1a). The AZCRA (investigation of the role of cytokines, chemokines and their receptors in the inflammatory process in asthma patients) study was approved by the local ethics committee of the Universitätsklinikum at the Friedrich-Alexander-Universität Erlangen-Nürnberg (FAU), Germany (Re-No. 315_20B). The study is registered in the German Clinical Trial Register (Deutsches Register Klinischer Studien: registration no. DRKS00023843). Informed consent was obtained from all participants included in the study. Asthmatic patients between 18 and 65 were recruited, and blood drawing was performed (Figure 1a). Additionally, control, non-atopic and non-asthmatic healthy subjects between 18 and 65 were recruited (Table 1). From the obtained whole blood samples, we isolated human PBMCs and cultured them with and without RV, and we analyzed the cells via flow cytometry and qPCR and the supernatants with ELISA at the end of the culture (Figure 1b).

### 2.2. Peripheral Blood Mononuclear Cell (PBMC) Isolation

PBMCs were isolated from EDTA blood with BioColl (Bio&Sell, Feucht, Germany) gradient, using Sepmate-50 Tubes (Stemcell Technologies, Cologne, Germany) according to manufacturer’s instructions. PBMCs were washed twice with RPMI 1640 without additives. The PBMC pellet was treated with ACK lysis (0.15 M NH_4_Cl (Carl Roth GmbH + Co. KG, Karlsruhe, Germany), 0.01 M KHCO_3_ (Carl Roth GmbH + Co. KG), 100 M Na_2_EDTA (Gerbu Biotechnik GmbH, Wieblingen, Germany), dissolved and sterile filtered in deionized H_2_O (pH = 7.2–7.4) to remove remaining erythrocytes. After isolation, PBMCs were counted and adjusted to a concentration of 1 × 10^6^ viable cells/mL in complete culture medium. To prepare the complete culture medium, RPMI 1640 (Gibco, Thermo Fisher Scientific, Waltham, MA, USA) medium was supplemented with 25 mmol/L HEPES (Gibco, Thermo Fisher Scientific). Additionally, 100 IU/mL penicillin, 100 μg/mL streptomycin, 50 μmol/L β-mercaptoethanol (Sigma-Aldrich, St. Louis, MO, USA), 1% L-glutamine (Anprotec, Bruckberg, Germany, 200 mmol/L), 1% MEM Vitamin (Sigma-Aldrich), 1% MEM nonessential amino acids (Gibco, Thermo Fisher Scientific), 1% sodium pyruvate (Gibco, Thermo Fisher Scientific) and 10% heat-inactivated FBS (Sigma-Aldrich) were added. The PBMCs were used for cell culture or RNA extraction of freshly isolated, untouched PBMCs that did not undergo cell culture with Qiazol Lysis Reagent (Qiagen, Venlo, The Netherlands).

### 2.3. Rhinovirus Infection

For rhinovirus infection, rhinovirus strain RV1B was used. RV1B is currently classified as RV-A species among other 79 rhinovirus serotypes that use intercellular adhesion molecule 1 as their cellular receptor [20]. RV1b was grown as previously described [21,22]. After PBMC isolation and before cell culture, some of the PBMCs were infected with rhinovirus suspension (500 μL/10^6^ cells) by shaking the cells for 1 h at 33 °C. After rhinovirus infection, the cells were washed with RPMI 1640 medium and cultured in the complete culture medium. The control condition was treated equally only without the presence of RV1B and cultured in the complete culture medium as well.

### 2.4. PBMC Cell Culture

For cell culture, previously infected cells were seeded at a concentration of 5 × 10^5^ cells in 0.5 mL complete culture medium on a 48-well plate (Greiner Bio-one, Frickenhausen, Germany). Cell culture was performed for 4 days at 37 °C and 5% CO2. As a control for rhinovirus-infected cells, cells were cultured with cell culture medium without previous RV infection. Supernatants were collected for ELISA, and RNA was extracted from the cells using Qiazol Lysis Reagent (Qiagen) for quantitative real-time PCR (qPCR).

### 2.5. Flow Cytometry Analysis

For FACS analysis, cells were collected after 4 days of cell culture, transferred into FACS tubes and washed once with PBS. Cells were stained for live/dead cells with Zombie Aqua Fixable Viability Kit (Biolegend, San Diego, CA, USA) diluted 1:500 in PBS for 15 min at room temperature according to manufacturer’s protocol. Live/dead staining was stopped with FACS buffer (PBS EDTA Lonza with 2% FCS), and cells were centrifuged at 1500 rpm, 4 °C for 5 min. Subsequently, cells were treated with Human TruStain FcX (Biolegend) for 10 min at 4 °C to inhibit unspecific binding of the antibodies. After centrifugation, supernatant was removed, and the prepared antibody cocktail (Table 2) was added to the cells. The antibodies were diluted in FACS buffer, and 50 µL per sample were used to stain the cells for 30 min at 4 °C. After stopping the staining with FACS buffer, cells were centrifuged and afterwards fixed for intracellular staining with Foxp3/Transcription Factor staining buffer set (Cat: 00-5523-00, ebioscience, Invitrogen, Thermo Fisher Scientific) for 35 min. After centrifugation, the intracellular antibodies were applied in Perm Wash for 30 min. Cells were washed with Perm wash, resuspended with FACS buffer and measured on a FACS Canto II (BD Biosciences, Heidelberg, Germany). FACS analysis was done with Kaluza analysis V2.1 (Beckmann Coulter, Brea, CA, USA) for windows. The gating strategies for the flow cytometry analysis are shown in Supplementary Figure S1.

### 2.6. RNA Isolation and Quantitative Real-Time PCR

RNA from cells was isolated using Qiazol lysis reagent (Qiagen). cDNA was synthesized with the RevertAid First Strand cDNA Synthesis Kit (Thermo Fisher Scientific) according to the manufacturer’s protocol. The quantitative real-time PCR was performed with iTaq Universal SYBR Green Supermix (Bio-Rad Laboratories, Hercules, CA, USA) using the CFX-96 Real-Time PCR Detection System (Bio-Rad Laboratories). The melting temperature was analyzed from 65–95 °C in 0.5 °C increments at 5 s/step. The gene expression of the genes of interest was normalized using the housekeeping gene *hHPRT* (5′-TGA CAC TGG CAA AAC AAT GCA-3′, 5′-GGT CCT TTT CAC CAG CAA GCT-3′). Analysis of gene expression of *hTGF-β1*, *hTGF-βRI*, *hTGF-βRII* and *RV1b* was performed using the following primers (Eurofins, Ebersberg, Germany): *hTGF-β1* (5′-CAC GTG GAG CTG TAC CAG AA-3′, 5′-GAA CCC GTT GAT GTC CAC TT-3′), *hTGF-βRI* (5´-GGA CCA GTG TGC TTC GTC T-3′, 5′-CAA TGG TAA ACCTG AGC CAG AA-3′), *hTGF-βRII* (5′-TTT TCC ACC TGT GAC AAC CA-3′, 5′-GGA GAA GCA GCA TCT TCC AG-3′) and *RV1b* (5′-CCA TCG CTC ACT ATT CAG CAC-3′, 5′-TCT ATC CCG AAC ACA CTG TCC-3′)

### 2.7. ELISA

To analyze the production of TGF-β1 in the supernatant of cultured PBMC, the supernatant was collected. Samples were incubated for 10 min at room temperature with 1N HCL to activate inactive TGF-β. Subsequently, 1.2 N NaOH was added to neutralize the samples again. The samples were then used for human TGF-β1 ELISA (Cat: DY240-05, Duoset, R&D Systems, Wiesbaden, Germany) according to manufacturer’s protocol. For the determination of human IFNγ, we used the BD OptEIA Kit (Cat: 555142, BD Biosciences) according to manufacturer’s protocol.

### 2.8. Statistical Analysis

Statistical analysis and graph design was performed with GraphPad Prism version 9 for windows (GraphPad Software, San Diego, CA, USA). Differences between two groups were evaluated for significance by the Student’s two-tailed t-test for parametric data or the Mann–Whitney U-test for non-parametric data. Differences between three or more groups were evaluated for significance by the one-way ANOVA for parametric data or the Kruskal–Wallis test for non-parametric data. For correlation analysis of non-parametric data, the Spearman correlation was used. Significances are shown as * *p* ≤ 0.05, ** *p* ≤ 0.01 and *** *p* ≤ 0.001. Data are given as mean values ± S.E.M. The sample size between different parameters varies because of limited materials from some individuals.

## 3. Results

### 3.1. Increased TGF-βRII in PBMC of Asthmatic Subjects

As we wanted to investigate the role of TGF-β and its receptors TGF-βRI and TGF-βRII in this human study of adult asthmatics and healthy controls, we analyzed the gene expression of *TGF-βRI* and *II* in untouched, freshly isolated PBMCs from whole blood that were not cultured. In this study, the expression of *TGF-βRI* was only tendentially higher in asthmatic individuals compared to healthy controls (Figure 1c). In contrast, the mRNA expression of *TGF-βRII* was significantly upregulated in the asthmatic patients (Figure 1d).

### 3.2. RV Infection Inhibited TGF-β Release by Peripheral Blood Mononuclear Cells (PBMCs) from Control and Asthmatic Adult Subjects In Vitro

In this study, we first were interested in further cellular investigations on the TGF-β release during rhinovirus infection in PBMCs and, therefore, infected isolated PBMCs with rhinovirus (RV1B) and after infection cultured them for 4 days. The mRNA levels of the RV1b were comparable in control and asthmatic PBMCs (Figure 1e). In this setup, we measured the expression of *TGF-βRII* mRNA and found no significant regulation in the rhinovirus-infected conditions (Figure 1f). As the PBMCs can also be producers of TGF-β1, we measured the expression of *TGF-β1* mRNA in the control and rhinovirus-infected PBMCs. Here again, we did not see a differential regulation of *TGF-β1* mRNA production upon rhinovirus infection (Figure 1g). Interestingly, when we measured TGF-β1 protein production in the cell culture supernatants of these cells, we found a significant downregulation of TGF-β1 activated by acidic condition on RV-infected samples (Figure 1h). These results suggest a disparity between the mRNA expression of TGF-β1 by PBMCs and its actual release, consistent with our previous findings in children [19].

### 3.3. High Rhinovirus Load Was Associated with More TGF-βRII mRNA Expression and Induced Treg Immune Response

**Figure 2:**
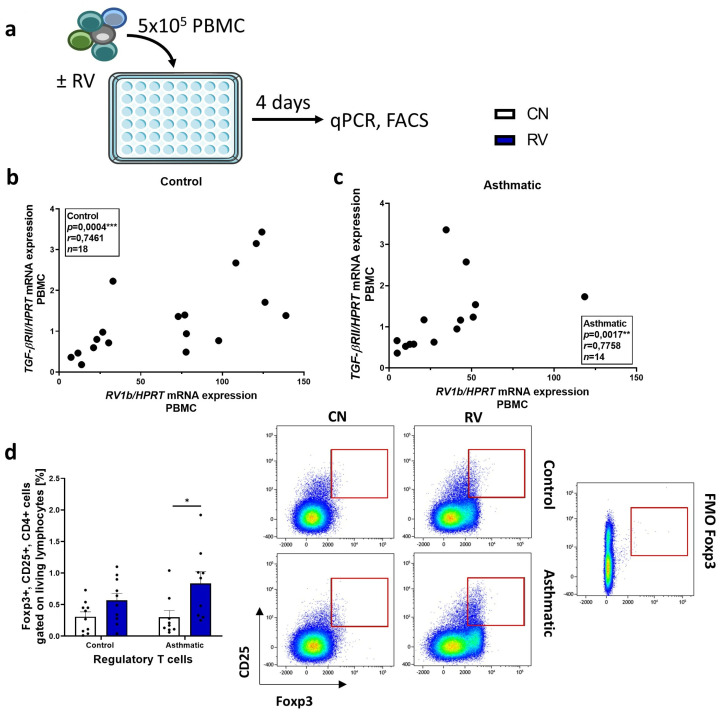
High rhinovirus load was associated with more TGF-βRII and induced Treg immune response. (**a**) Experimental design. (**b**) Correlation of *TGF-βRII/HPRT* expression in RV-infected PBMCs with the *RV1b/HPRT* mRNA expression in controls. (**c**) Correlation of *TGF-βRII/HPRT* expression in RV-infected PBMCs with the *RV1b/HPRT* mRNA expression in asthmatics. (**d**) Flow cytometry analysis of Foxp3+ CD25+ CD4+ Tregs in CN and RV condition. A representative dot plot of each group and the FMO control is shown (*n* = 10, 9, 10, 9). Data are shown as Mean+SEM. * *p* < 0.05, ** *p* < 0.01, *** *p* < 0,001.

As we did not see differences in *TGF-βRII* mRNA expression between the CN and RV conditions, but rather variations between individuals, we assumed that there might be other factors influencing *TGF-βRII* gene expression. We correlated it with the *RV1b/HPRT* mRNA expression and found a significant positive correlation between these parameters in controls and asthmatic patients (Figure 2a–c). In the RV-infected PBMCs, we additionally investigated the immune response following the infection. Therefore, we stained for regulatory T cells in CN and RV conditions. Here, we saw that rhinovirus infection boosted Treg response, especially in asthmatic individuals (Figure 2d). The FMO control for Foxp3 is shown in the representative dot plots. Taken together, higher TGF-βRII signaling was associated with more virus mRNA and RV induced a Treg-mediated immune response.

### 3.4. Production of TGF-β in T Cells Is Not Affected by Rhinovirus Infection

Next, we were wondering which cells might be responsible for the reduced TGF-β1 production, and we analyzed the PBMCs via flow cytometry. We stained the cells with TGF-β antibody used in surface or intracellular staining, to determine the amount of TGF-β on the cell surface and in the cytosol. Here, we divided the cells into CD3+ T cells and CD3−non-T cells. In both cell populations, we found a slight reduction in surface-bound TGF-β. Additionally, asthmatic patients had a tendentially higher percentage of surface TGF-β in the control condition as compared to healthy individuals (Figure S2a,b). In the intracellular compartment, we did not see a clear regulation of TGF-β upon RV infection. There were also no apparent differences between healthy controls and asthmatics in T cells as well as non-T cells (Figure S2c,d).

### 3.5. Rhinovirus Infection Downregulated GARP-Bound TGF-β on the Cell Surface of NK Cells

**Figure 3:**
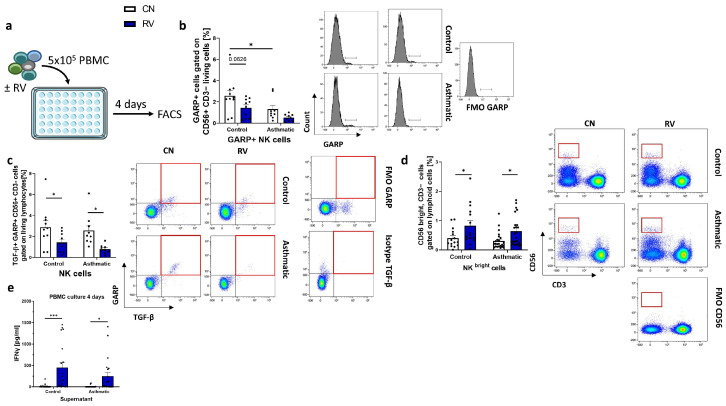
NK cells produce less TGF-β upon rhinovirus infection. (**a**) Experimental design. (**b**) Flow cytometry analysis of GARP-positive NK cells (*n* = 10, 9, 10, 9). (**c**) Flow cytometry analysis of intracellular TGF-β+ GARP+ NK cells. A representative dot plot for each group is shown (*n* = 10, 9, 10, 9). (**d**) Flow cytometry analysis of CD56 bright NK cells (*n* = 15, 24, 15, 24). (**e**) IFNγ ELISA of supernatants from CN and RV conditions (*n* = 19, 22, 19, 22). Data are shown as Mean+SEM. * *p* < 0.05, *** *p* < 0.001.

The flow cytometry analysis of T cells and non-T cells did not show significantly reduced TGF-β production, so we reasoned that there has to be a smaller subset of cells responsible for the lack of TGF-β upon RV infection. In addition, we also wanted to investigate the possibility that the TGF-β might still be bound to the membrane of immune cells and cannot be released. Therefore, we stained for surface expression (Figure 3a) of the GARP protein. Surprisingly, we discovered that NK cells showed GARP expression on their surface. In this population we found reduced expression of GARP in the RV-infected condition (Figure 3b). Moreover, asthmatics had a significantly lower expression of GARP in the CN condition compared to controls. Next, we stained for co-expression of GARP and TGF-β. We analyzed this population in the control and rhinovirus-infected condition and found that it was significantly reduced upon infection (Figure 3c). These results suggest that NK cells are crucially involved in the reduction of TGF-β release during rhinovirus infection of PBMC. Furthermore, we report that not only Tregs express the LAP-TGF-β-GARP complex, but also NK cells.

### 3.6. Reduction of TGF-β during Rhinovirus Infection Promotes CD56 High NK Cells and IFNγ Production in Healthy Controls and Asthmatics

We reasoned that suppression of TGF-β release and presentation on the cell membrane by NK cells might boost the antiviral NK cell response of the host, as NK cells exert important functions in the immune answer. Therefore, we analyzed the NK cell response upon RV infection in cultured PBMCs via flow cytometry. In this study, we found an upregulation of CD56 bright NK cell population upon RV infection (Figure 3d). This population is known to be important for the production of antiviral cytokines [23]. Consistent with an induction of this NK population, we found increased IFNγ levels in the supernatant of RV-infected PBMCs (Figure 3e). These results suggest that the reduction of TGF-β during rhinovirus infection might promote IFNγ producing CD56 high NK cells in healthy controls and asthmatics.

## 4. Discussion

In summary, our results show that asthmatic individuals have higher expression of TGF-βRII. In controls and asthmatics, TGF-β release is drastically reduced in rhinovirus-infected PBMCs due to decreased production by NK cells.

Our previous publication on the PreDicta cohort showed similar results in preschool children [19]. Rhinovirus infection reduced TGF-β production markedly, while in contrast to our adult participants, there was an induction of *TGF-βRII* mRNA levels in the RV-infected cells. The adult asthmatics showed higher *TGF-βRII* gene expression in untouched PBMC. It might be that asthmatic subjects, capture free TGF-β via binding to TGF-βRII without exerting any signal transduction, as TGF-βRI is not differentially regulated. On the other hand, this could represent a better responsiveness to TGF-β1 binding and could therefore drive tissue remodeling in the asthmatic patients with higher receptor expression [24]. Moreover, TGF-β receptors are also known to undergo various posttranslational modifications that might alter the receptor activity [25]. Further investigations in this direction would be needed.

In asthmatic patients, we found a high variability of TGF-β on the surface of T cells, which is probably dependent on disease severity and asthmatic symptoms at the timepoint of the visit. Nevertheless, in the T cell and non-T cell populations, we could not detect significant differences. An enlargement of the patient numbers might allow for a more detailed analysis.

Previous studies showed that regulatory T cells have the special ability to bind the LAP-TGF-β complex to their cell surface via the GARP transmembrane protein. GARP is highly expressed on activated Tregs and is important for maintaining the Treg function and homeostasis [26]. It was shown that TCR activation induced GARP surface expression on Tregs but not on Th cells [16]. Both T cell subsets are able to secrete latent TGF-β, but only Tregs can capture it with GARP [27]. So far GARP was thought to be nearly exclusively expressed on Tregs [28]. In this study, we show that NK cells are also able to express LAP-TGF-β-GARP complex on their surface.

TGF-β1 is a powerful immunoregulatory and immunosuppressive cytokine. It exerts many different functions, including the suppression of IFN Type I released from alveolar macrophages [29]. It is known to impair the differentiation and proliferation of antiviral, cytotoxic CD8+ T cells [30]. TGF-β is a regulator of NK cell homeostasis and prevents a dysregulated NK cell response [31]. Furthermore, NK cells are one of the main sources of TGF-β production, comparable to monocytes [32]. The literature states that TGF-β can suppress IFN-γ production via SMAD signaling by alteration of Tbet activity [33]. Recently, it was also found that severe COVID-19 patients lack NK cells as compared to mild cases. Severe cases had a TGF-β-imprinted gene regulation in NK cells and showed reduced cytotoxic activity by lacking IFN-γ production and via the suppression of Tbet. An untimely early TGF-β response to control the antiviral immune answer led to impaired NK cell activation and therefore more severe disease [34].

Additionally, TGF-β inhibits mTOR-dependent metabolic activity in NK cells stimulated by IL-15 [35]. TGF-β also induces the conversion of NK cells into ILC1, which are predominantly tissue resident and only have limited cytotoxic abilities [36,37]. These different properties of TGF-β suppress NK cell cytotoxic activity and impair the antiviral response [38]. Taken together, these findings suggest that high TGF-β levels are not beneficial for the immune response against rhinovirus, and NK cells might consistently downregulate its production.

In the context of rhinovirus infection, it is also known that TGF-β increases RV load, and therefore, it could also support the antiviral response if immune cells produce less TGF-β [39]. Bedke et al. show a suppressive effect of TGF-β on the production of IFN-β by primary bronchial epithelial cells infected with rhinovirus. They also find a reduction of IFN-λ1 in TGF-β-treated condition. When they applied neutralizing anti-TGF-β antibody, the virus replication was found to be suppressed, supporting previous findings [40].

This study is only at the beginning to understand the involvement of TGF-β in anti-rhinovirus response. There is the need of an enlarged study with more patients to confirm these findings. NK cell sorting and single-cell sequencing of peripheral NK cells would additionally strengthen the novel findings we present here.

In conclusion, high TGF-β1 levels during rhinovirus infection impair the immune response and lead to pro-viral effects in the periphery. This study shows that NK cells express the latent-TGF-β-GARP complex on their cell surfaces. Furthermore, they are crucially involved in downregulation of TGF-β production during antiviral immune response to support NK cell cytotoxicity and function.

## Figures and Tables

**Table 1:** Clinical characteristics of the control and asthmatic individuals in the human AZCRA study.

ID	Gender	Age	Asthma Severity ^1^	Treatment	Asthma Symptom Control (GINA 2020)
C01	F	34	**/**	**/**	**/**
C02	F	33	**/**	**/**	**/**
C03	F	29	**/**	**/**	**/**
C04	F	44	**/**	**/**	**/**
C05	M	62	**/**	**/**	**/**
C06	M	64	**/**	**/**	**/**
C07	M	26	**/**	**/**	**/**
C08	F	23	**/**	**/**	**/**
C09	F	63	**/**	**/**	**/**
C10	F	53	**/**	**/**	**/**
C11	M	55	**/**	**/**	**/**
C12	M	21	**/**	**/**	**/**
C13	F	22	**/**	**/**	**/**
C14	M	31	**/**	**/**	**/**
C15	M	25	**/**	**/**	**/**
C16	M	43	**/**	**/**	**/**
C17	M	30	**/**	**/**	**/**
C18	F	28	**/**	**/**	**/**
C19	M	24	**/**	**/**	**/**
A01	M	28	Mild persistent	Steroid + non-steroid	Well controlled
A02	F	33	Mod. Persistent	Steroid + non-steroid	Partly controlled
A03	M	63	Mild persistent	Steroid + non-steroid	Well controlled
A04	M	53	Mild persistent	Steroid + non-steroid	Partly controlled
A05	M	57	Mild persistent	Steroid + non-steroid	Well controlled
A06	F	47	Mild persistent	Steroid + non-steroid	Well controlled
A07	F	40	Mod. Persistent	Steroid + non-steroid	Partly controlled
A08	M	60	Intermittent	None	Well controlled
A09	F	44	Mild persistent	Steroid + non-steroid	Well controlled
A10	F	51	Mild persistent	Steroid + non-steroid	Partly controlled
A11	M	26	Intermittent	None	Partly controlled
A12	F	56	Intermittent	None	Well controlled
A13	M	31	Intermittent	Steroid + non-steroid	Partly controlled
A14	M	24	Mild persistent	Steroid	Partly controlled
A15	F	47	Intermittent	Steroid + non-steroid	Well controlled
A16	F	26	Intermittent	None	Partly controlled
A17	F	54	Intermittent	Steroid + non-steroid	Well controlled
A18	F	47	Mod. Persistent	Steroid + non-steroid	Partly controlled
A19	F	24	Mod. Persistent	Steroid + non-steroid	Uncontrolled
A20	M	23	Intermittent	None	Well controlled
A21	M	41	Mod. Persistent	Steroid + non-steroid	Partly controlled
A22	F	64	Mod. Persistent	Steroid + non-steroid	Partly controlled
A23	F	62	Unknown	None	Unknown
A24	F	52	Intermittent	Steroid + non-steroid	Partly controlled
A25	M	48	Intermittent	Steroid + non-steroid	Partly controlled
A26	M	35	Intermittent	Steroid + non-steroid	Well controlled
A27	F	52	Unknown	Steroid + non-steroid	Well controlled
A28	F	54	Mod. Persistent	Steroid + non-steroid	Partly controlled

^1^ According to GINA 2005 Guidelines. Intermittent: Symptoms less than once a week, brief exacerbations, nocturnal symptoms not more than twice a month, normal lung function between episodes. Mild Persistent: Symptoms more than once a week but less than once a day, nocturnal symptoms more than twice a month but less than once a week, normal lung function between episodes. Moderate Persistent: Symptoms daily, exacerbations may affect activity and sleep, nocturnal symptoms at least once a week, 60% < FEV1< 80%; predicted OR 60% < PEF < 80% of personal best.

**Table 2:** Flow cytometry antibodies.

Antibody	Company	Catalog Number	RRID
BV421 anti-human CD56	BD Biosciences	562752	AB_2732054
APC-Fire anti-human CD3	Biolegend	300470	AB_2629689
PerCP/Cyanine 5.5 anti-human GARP	Biolegend	352513	AB_2734371
PE/Cyanine7 anti-human CD25 antibody	Biolegend	302612	AB_314282
Alexa Fluor 647 anti-human FOXP3 antibody	Biolegend	320214	AB_492984
PE anti-human LAP recombinant	Biolegend	364403	AB_2910407
PE anti-mouse IgG1, κ	Biolegend	400113	AB_326435
Zombie Aqua Fixable Viability Kit	Biolegend	423101	

## Data Availability

The datasets generated for this study can be accessed upon request to the corresponding author.

## Supplementary Materials

The following supporting information can be downloaded at: https://www.mdpi.com/article/10.3390/cells12010129/s1, Figure S1: Flow cytometry analysis, Figure S2: TGF-β production in T cells is not affected by rhinovirus infection.
