# Supplementary material for: Rhinovirus Suppresses TGF-β-GARP Presentation by Peripheral NK Cells
Source: Cells. 2022 Dec 28;12(1):129. doi: 10.3390/cells12010129 (PMC9818541; doi:10.3390/cells12010129)
Supplement: Supplementary file 1 [file cells-12-00129-s001.zip › cells-2050447-supplementary.pdf]

cells

Supplementary materials

Rhinovirus Suppresses TGF-β-GARP Presentation by Peripheral NK Cells

Susanne Krammer [1], Zuqin Yang [1], Hannah Mitländer [1], Janina C. Grund [1], Sonja Trump [1], Susanne Mittler [1], Sabine Zirlik [2] and Susetta Finotto [1,*]

[1] Department of Molecular Pneumology, Friedrich-Alexander-Universität (FAU) Erlangen-Nürnberg, Universitätsklinikum Erlangen, 91054 Erlangen, Germany[2] Department of Medicine 1, Friedrich-Alexander-Universität (FAU) Erlangen-Nürnberg, Universitätsklinikum Erlangen, 91054 Erlangen, Germany

* Correspondence: susetta.finotto@uk-erlangen.de ; Tel.: +49-9131-85-35883

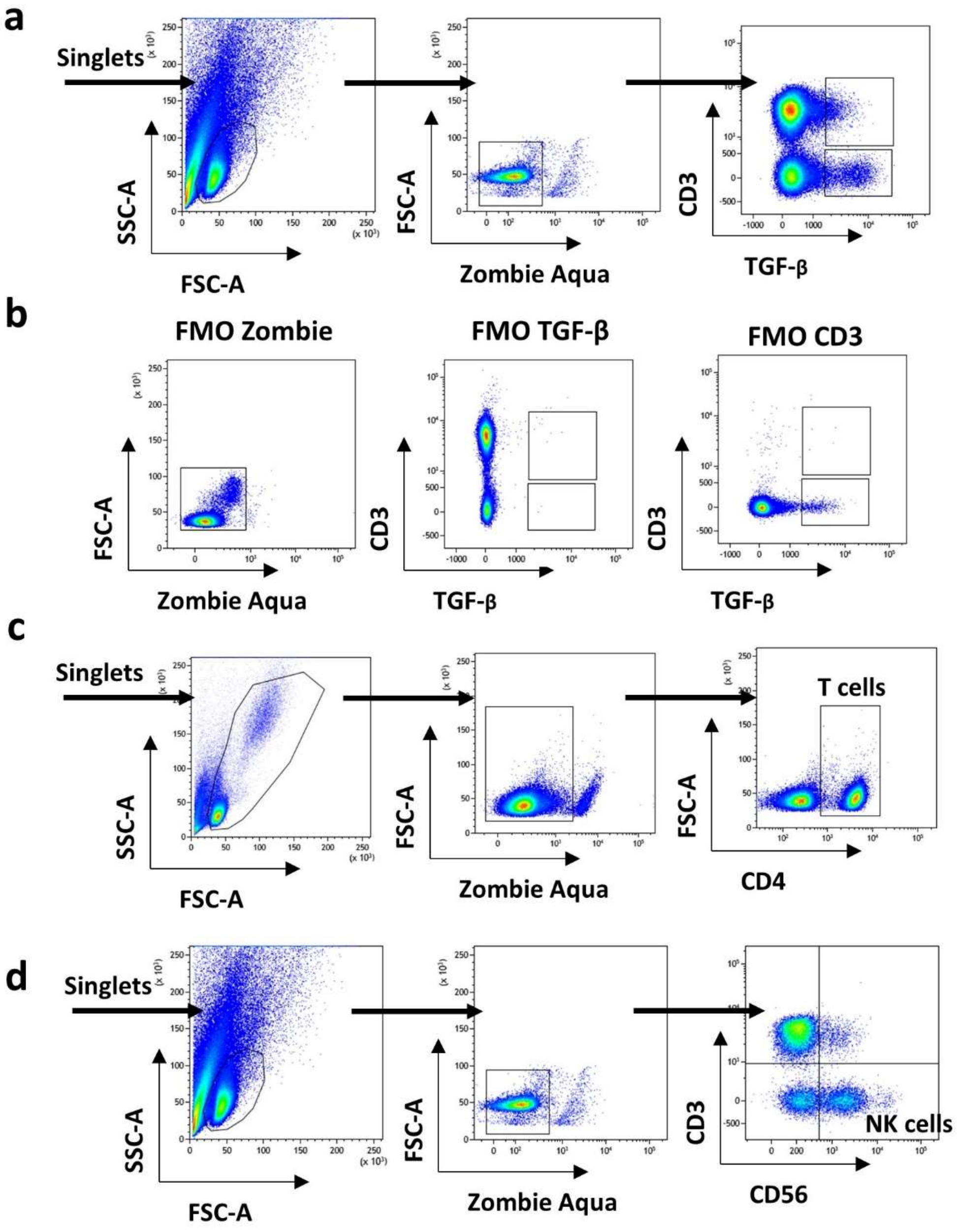

Figure S1. Flow cytometry analysis**.** (**a**) Gating strategy for TGF-β analysis on CD3+ and CD3− cells. (**b**) FMO controls for the flow cytometry analysis of TGF-β on T cells and non-T cells. (**c**) Gating strategy for analysis of Tregs. (**d**) Gating strategy for TGF-β and GARP analysis on NK cells.

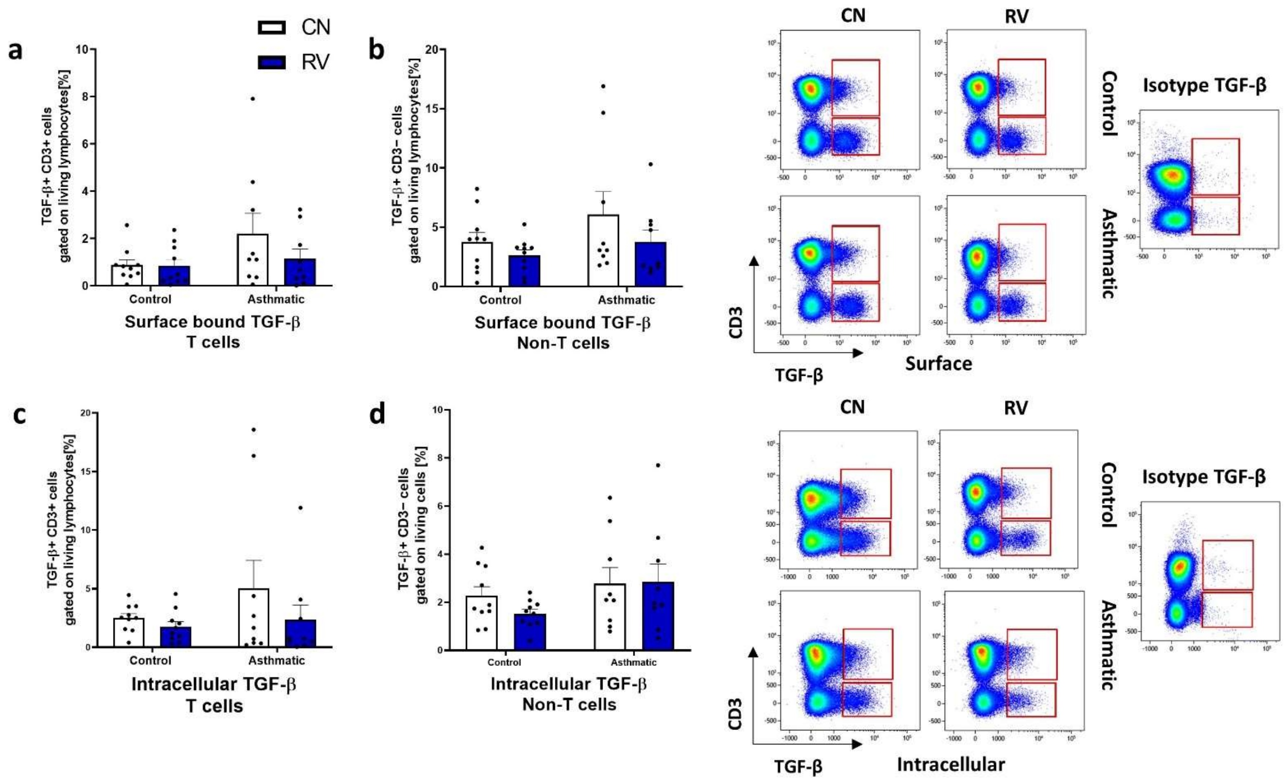

Figure S2. TGF-β production in T cells is not affected by rhinovirus infection. (**a**) Flow cytometry analysis of surface bound TGF-β on T cells. (**b**) Flow cytometry analysis of surface bound TGF-β on CD3− non-T cells. A representative dot plot for each group is shown. (**c**) Flow cytometry analysis of intracellular TGF-β in T cells. (**d**) Flow cytometry analysis of intracellular TGF-ß in CD3− non-T cells. A representative dot plot for each group is shown. Data (*n*=10, 9, 10, 9) is shown as Mean+SEM.
